# Bayesian trial of adalimumab versus secukinumab for children with juvenile idiopathic arthritis associated uveitis or chronic anterior uveitis

**DOI:** 10.1186/s12969-025-01107-1

**Published:** 2025-05-19

**Authors:** Athimalaipet V Ramanan, Andrew D Dick, Thomas Jaki, Gianmarco Caruso, David S Robertson, Ashley P Jones, Ben Hardwick, Sian Drake, Balini Balasubramaniam, Coziana Ciurtin, Ivan Foeldvari, Elke O Kreps, Alice Leahy, Kristina May, Pierre Quartier, Matthieu P Robert, Gabriele Simonini, Catherine Guly, Michael W Beresford

**Affiliations:** 1 Bristol Royal Hospital for Children, Upper Maudlin Street, Bristol, BS2 8BJ UK; 2 Translational Health Sciences, University of Bristol, Bristol, UK; 3 Bristol Medical School, University of Bristol, Bristol, UK; 4 NIHR Moorfields Biomedical Research Centre, UCL Institute of Ophthalmology, London, UK; 5 University of Regensburg, Regensburg, Germany; 6 University of Cambridge, Cambridge, UK; 7 Liverpool Clinical Trials Centre, The University of Liverpool, Liverpool, UK; 8 University Hospitals Birmingham NHS Foundation Trust, Birmingham, UK; 9 Centre for Adolescent Rheumatology, University College London, London, UK; 10 Hamburg Center for Pediatric and Adolescent Rheumatology, Hamburg, Germany; 11 Ghent University Hospital, Ghent, Belgium; 12 University Hospital Southampton NHS Foundation Trust, Southampton, UK; 13 Université Paris Cité, Paris, France; 14 RAISE Reference centre, Pediatric Immunology-Hematology and Rheumatology Unit, Necker-Enfants malades hospital, APHP, Paris, France; 15 OPHTARA reference center, Ophthalmology Department, Necker-Enfants malades hospital, APHP, Paris, France; 16 Borelli Center, UMR 9010, CNRS-SSA-ENS Paris Saclay-Université Paris Cité, Paris, France; 17 Rheumatology unit, ERN ReCONNET center, FIRENZE and NEUROFARBA Department, Meyer Children’s Hospital IRCCS, University of Florence, Florence, Italy; 18 Bristol Eye Hospital, Bristol, UK; 19 Department of Paediatric Rheumatology, Alder Hey Children’s NHS Foundation Trust, Liverpool, UK; 20 Institute of Life Course and Medical Sciences, University of Liverpool, Liverpool, UK

**Keywords:** Juvenile idiopathic arthritis, Uveitis, Bayesian prior, Clinical trial, Biologics

## Abstract

**Background:**

Juvenile idiopathic arthritis (JIA)-associated uveitis and chronic anterior uveitis in children may result in permanent sight loss. Currently, the only licensed and approved treatment for JIA-uveitis is adalimumab. However, even in patients where adalimumab may be initially effective, therapeutic response may subside for example, due to neutralising drug antibodies. Further treatment options are necessary to prevent sight loss in children with uveitis. Interleukin 17 is elevated in uveitis. Inhibition of interleukin 17 ameliorates inflammation in mouse models of uveitis. Secukinumab, an antibody which neutralizes interleukin 17 A, has been shown to be partially effective in adult uveitis. The objective of the Bayesian consensus meeting was to quantify prior expert opinion about the potential utility of secukinumab in treatment of uveitis in JIA.

**Methods:**

Nine international experts in paediatric rheumatology, paediatric ophthalmology and/or paediatric uveitis took part in a structured Bayesian prior elicitation meeting.

**Results:**

The final consensus was that adalimumab is expected to yield a higher response rate than secukinumab (mean 0.67 vs. 0.55). The uncertainty in the response rate on secukinumab is somewhat larger than for adalimumab. The equivalent sample size for the prior distribution of adalimumab is 15.7 and 13.1 for secukinumab. The decisions based on the combined evidence would still be driven by the trial data, yet substantial enhancement of the power of the study can be expected by adding information from the equivalent of almost 30 patients.

**Conclusions:**

The Bayesian analysis adds substantial enhancement of the power of the study and supports a head-to-head trial of adalimumab and secukinumab for JIA-associated uveitis and chronic anterior uveitis.

**Trial registration:**

ISRCTN 12,427,150 Registration date 14/02/2023. EudraCT 2022-003068-26 Registration date 07/09/2022.

**Supplementary Information:**

The online version contains supplementary material available at 10.1186/s12969-025-01107-1.

## Introduction

Juvenile idiopathic arthritis (JIA) is the most common rheumatic disease in children and young people. Approximately 1 in 1000 children in the UK develop JIA per annum. Although both genders are affected, JIA is more common in girls. Amongst those children with JIA, around 15–25% are at risk of intra-ocular inflammation known as uveitis [1, 2]. In children, uveitis is typically asymptomatic in the initial stages of mild-moderate inflammation. Children with active uveitis are at significant risk of ocular complications leading to sight loss [3–5]. Previously, JIA-associated uveitis was managed with topical corticosteroid eye drops alone but this was mostly inadequate to control uveitis; furthermore, their long-term use led to a high risk of cataract and glaucoma.

The majority of children with JIA uveitis are now treated with systemic methotrexate (MTX) +/- corticosteroid eye drops [6, 7] but over 40% require a biologic agent due to inadequate control of uveitis [8, 9]. The SYCAMORE trial demonstrated that adalimumab with methotrexate is effective in controlling JIA-associated and chronic anterior uveitis, but the treatment was ineffective in 27% of patients [10]. Adalimumab may be effective initially and then lose effect in some children due to drug antibodies [11, 12]. There are multiple biologic treatments licensed for arthritis but only adalimumab is licensed for uveitis and further treatment options are necessary to prevent sight loss from uveitis in JIA. Adalimumab is currently standard of care and hence will serve as our benchmark in this exercise.

Interleukin 17 is known to be elevated in the serum of uveitis patients and inhibition of interleukin 17 ameliorates inflammation in mouse models [13, 14]. Secukinumab is an antibody which neutralizes interleukin 17 A. Intravenous secukinumab has been shown to be effective inducing remission and reduction in topical and systemic corticosteroids use in adult uveitis in a phase 2 clinical trial [15] compared to subcutaneous dosing. Subcutaneous delivery in three adult randomised controlled trials (RCT) did not reach primary end points but did support a reduction in concomitant immunosuppressive medication [16]. Of note, there have been some reports of a low incidence of new uveitis and flares of uveitis in patients with ankylosing spondylitis treated with intravenous or subcutaneous secukinumab [17].

There have been no published paediatric RCT in JIA-associated uveitis or chronic anterior uveitis looking at treatment with secukinumab. It is unlikely that a phase III RCT in JIA-associated uveitis or chronic anterior uveitis is feasible at this time due to the rarity of patients and therefore the time it will take to complete such a trial. To urgently gather and present reliable evidence for the safety and efficacy of secukinumab treatment in JIA-associated uveitis or chronic anterior uveitis, a Phase II trial randomising 40 patients aged 2–18 years to either adalimumab or secukinumab will be conducted (the TURTLE Trial EudraCT number: 2022-003068-26). In the first part of the study, 10 patients refractory to adalimumab treatment will be recruited to receive secukinumab treatment. It was believed that higher drug concentrations are required to achieve efficacy in the eye, so a higher dose was chosen for this study. This higher dose is based on the licence for secukinumab in paediatric psoriasis patients which states that the dose can be increased to 300 mg. Subcutaneous secukinumab was chosen instead of intravenous as it was more pragmatic and less burdensome on participants and their families. If at least three out of the ten patients recruited show a response to treatment, then we will proceed to the randomisation stage. Response to treatment is defined as per the Standardisation of Uveitis Nomenclature (SUN) criteria [18] as a 2-step decrease in the level of inflammation (anterior chamber cells) or decrease to zero between baseline (prior to trial treatment initiation) and after 12 weeks of treatment. In routine practice patients would be seen in joint ophthalmology and rheumatology clinics and the SUN criteria would be reported by the ophthalmologist. It is unlikely based on this sample size that there would be sufficient power to reliably detect a statistically significant difference between treatments. However, this will be a large enough sample size to be clinically relevant to inform treatment decisions. With this in mind, we propose a Phase II trial to be performed using a Bayesian trial design.

Bayesian designs are an innovative approach to clinical studies and particularly valuable in small, but potentially diverse patient populations [19]. The Bayesian approach will allow publication of the combined prior evidence and the observed evidence in the trial. It is essential as a first step in a Bayesian trial to carefully record available knowledge before the new RCT begins. We followed the approaches used previously in a proposed trial of adalimumab versus pamidronate for children with chronic non-bacterial osteomyelitis / chronic recurrent multi-focal osteomyelitis (CNO/CRMO) [20], a trial of mycophenolate mofetil for childhood polyarteritis nodosa [21] and a proposed trial of methotrexate versus mycophenolate mofetil in juvenile localised scleroderma [22].

In order to determine the Bayesian prior, a face-to-face meeting of international paediatric Rheumatology and Ophthalmology experts experienced in treating JIA-associated uveitis or chronic anterior uveitis was convened. We used behavioural aggregation to establish consensus prior distributions that will underpin the future juvenile idiopathic arthritis associated uveitis or chronic anterior uveitis trial. The outcomes from this process are presented here.

## Methods

### Establishing a group of experts to determine consensus prior opinion

A consensus meeting was held over two days which brought together four experts from the United Kingdom and five experts from across Europe. Invitations were initially sent out to 29 experts across the United Kingdom and Europe and both rheumatologists and ophthalmologists were invited. Experts needed to have had recent experience in treating JIA-associated uveitis, had no connection to the planned trial and be willing to take part in the expert elicitation meeting. Based on responses to invitations, all nine experts who volunteered were invited to take part in the face-to-face meeting. The purpose of the meeting was to assess expert consensus but not to confirm efficacy of secukinumab. This meeting was held before the trial began rather than at the conclusion of the first stage of the trial. The idea of using elicited information is to strengthen the trial results by augmenting them with expert opinion. Using data from the study to determine how much to strengthen would lead to potential double counting information (rather than augmenting with additional information) and should therefore be avoided.

### Process of Establishing consensus prior opinion

The meeting took place in London, UK on the 10th and 11th of October 2023 and proceeded according to the agenda listed in Table 1. The first day focussed on describing the planned study, the study end points and a detailed review of the existing evidence around treatment options for uveitis associated with JIA. Results of available evidence and scientific rationale and pre-clinical evidence behind the use of IL-17 A inhibition in uveitis were also presented at the meeting Additionally, the statistical framework that would be used was introduced and the approach to elicitation discussed. Finally, a mock elicitation exercise using a fictitious example was held to ensure the process and the questions asked was fully understood.

**Table 1:** Activities comprising the consensus meeting and the time dedicated to each

Time allocation (minutes)	Activity
**Day 1**	
30	An introduction to the study and study endpoints
45	Review of current treatments, trial treatments, and associated current evidence base
105	Introduction to Bayesian methods
**Day 2**	
30	Recap from Day 1
60	Formal elicitation exercise
30	Presentation of the individual priors
75	Group discussion to reach consensus
15	Presentation of consensus priors

The second day was devoted to the formal elicitation process, structured so that the opinions of individuals were established before attempting to reach a consensus. This structure was adopted to reduce the risk of experts being unduly influenced by overconfident group members or those with strong personalities. Three facilitators (TJ, GC, DR) with statistical training in Bayesian methodology were on hand throughout to facilitate. Each expert was asked to complete a structured questionnaire (see Supplementary materials). They then had a one-to-one meeting with a facilitator during which the answers provided were visualised (and potentially adjusted) to ensure they reflected the experts’ opinion. Neither the trial Chief Investigators (AVR, MWB) nor the trial lead ophthalmologists (CG, AD) took part in the questionnaires. After each expert had completed their one-to-one meeting the facilitators summarised answers and a structured discussion ensued, moderated by the trial Chief Investigators (AVR, MWB) and a statistical facilitator (TJ).

### Approach for Establishing bayesian prior distributions

The goal of this elicitation exercise was to characterise the current understanding about the effectiveness of adalimumab and secukinumab and to construct informative Bayesian prior distributions to be combined with future trial data. In the following we followed the statistical methodology described in [21] and applied in [22].

### Defining the quantities to be elicited

The elicitation focused on the planned primary endpoint of the study. This is defined as response to treatment as per SUN criteria as a 2-step decrease in the level of inflammation (anterior chamber cells) or decrease to zero between baseline (prior to trial treatment initiation) and after 12 weeks of treatment.

This endpoint was used in the individual elicitations and formed the basis for the consensus discussion. During the consensus discussion, which included both ophthalmology and rheumatology experts, however, the experts suggested that a slightly modified endpoint might result in a more meaningful trial. The proposal was to consider additionally a decrease to 0.5 (instead of 0) already as response. Rather than deciding about which endpoint the future stage of the trial should adopt, an ad-hoc decision was made to also obtain a consensus opinion on the modified endpoint as well as the current definition of response, to enable it to be used should investigators decide to do so.

### Statistical model

Prior distributions were found for the response rate of adalimumab (ADA) and secukinumab (SEC), p_ADA_ and p_SEC_ while the log odds ratio, defined as

$$\:\theta\:=log\left(\frac{{p}_{SEC}\left(1-{p}_{ADA}\right)}{{p}_{ADA}\left(1-{p}_{SEC}\right)}\right),$$

was used to compare the two treatments. Positive values of θ imply that SEC is superior to ADA and conversely for negative values.

Following [21] the parameters p_ADA_, and θ were elicited directly from the experts. A beta distribution was used to model p_ADA_ while a normal distribution was used for θ. The prior distribution of p_SEC_ was numerically derived from the distributions of p_ADA_, and θ. This was done so that the p_ADA_ and θ could be treated as independent of each other.

### Establishing expert opinion

We first asked each expert to provide initial responses to the questions which subsequently were discussed in a one-to-one meeting with a statistician. An R Shiny application [23] was used for visualizing the prior distributions implied by the initial responses and alterations were made as required until the resulting distributions adequately reflected the expert’s opinion.

These individual prior distributions were then presented to all experts to initiate a discussion of the opinions. The goal was to arrive at a consensus prior distribution via behavioural aggregation following the Sheffield Elicitation Framework [24]. The facilitators of the group discussion highlighted apparent discrepancies between distributions to stimulate discussion.

Should, against expectation, no consensus be achieved, the plan was to use mathematical aggregation of the individual experts’ distribution using equal weight for each.

## Results

**Fig. 1:**
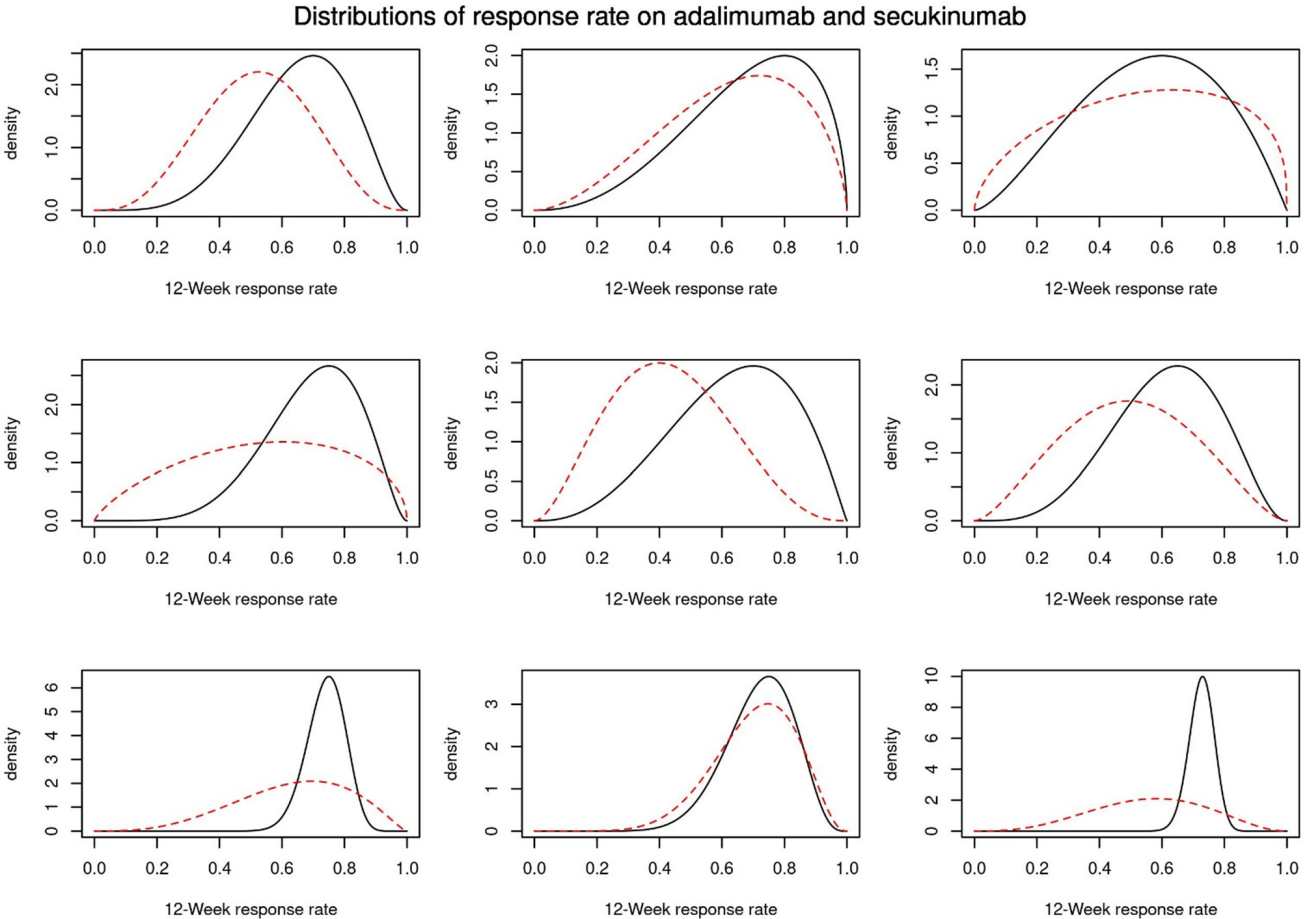
Individual elicited prior distributions. The solid black line corresponds to the prior distribution of the response rate of patients on adalimumab while the dashed red line shows the prior for secukinumab

The first outcome of the elicitation meeting were prior distributions from each expert (Fig. 1). These were used to stimulate the consensus discussion. From these distributions one can see a diverse opinion about each of the two treatments. However, in general experts were more confident in the response rate for adalimumab than secukinumab. Some experts had little uncertainty that around 60–80% of patients respond to adalimumab. Additionally, the experts agreed that, a priori, the response rate of secukinumab is expected to be no better than adalimumab.

### Consensus opinion

The individual prior distributions were used to seek a consensus opinion amongst the experts.

**Fig. 2:**
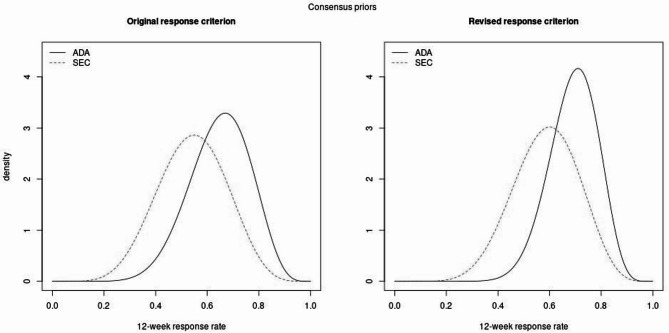
Consensus distribution for the original and revised definition of response. The solid black line corresponds to the prior distribution for the response rate on adalimumab while the dashed red line is for the prior for secukinumab

During the ensuing discussions a modification to the response criterion (two-step reduction in anterior chamber cell SUN score at 12 weeks) was suggested and it was decided to obtain two consensus prior distributions (Fig. 2). The final consensus for the original response definition was that adalimumab is expected to yield a higher response rate than secukinumab (mean 0.67 vs. 0.55). The uncertainty in the response rate on secukinumab is somewhat larger than for adalimumab although much less so than observed in some of the individual prior distributions.

Comparing the two definitions of response, the general patterns were very similar with the proposed revised definition of response yielding higher response rates for both treatments as expected. Additionally, the broader experience in using adalimumab in patients with JIA-associated uveitis demonstrated that the certainty in adalimumab is increased under the revised response definition while it is virtually unchanged for secukinumab.

## Discussion

The study objective was to perform a Bayesian prior elicitation exercise with international experts in paediatric rheumatology and paediatric uveitis to quantify the current understanding of the efficacy of adalimumab and secukinumab in JIA-associated uveitis and chronic anterior uveitis.

The experts were asked about the likelihood of adalimumab and secukinumab leading to a two-step reduction in anterior chamber cell SUN score at 12 weeks in children with JIA associated uveitis and chronic anterior uveitis.

The distribution of response to secukinumab in the 9 experts is different compared to adalimumab. This is due to the differing experiences of the different experts. Despite the difference in them, however, together they do provide crucial information about the expected activity of secukinumab. For example, it is clear that experts believe that the response rate is not lower than 20% and not higher than 80%. This information will help with more precise estimation of the true effect once trial data are added in the final analysis.

### Impact of consensus opinion on planned clinical trial

For simplicity we will now discuss the impact of the prior distribution on the Bayesian analysis of a future trial only in the context of the original definition of response. Interpretation of the results are similar for the revised definition of response.

The distributions obtained via consensus from the experts clearly show that there exists some agreed understanding of the likely efficacy of the two treatments prior to conducting an RCT based on treating patients with these biologics for JIA-associated uveitis, and other related indications, including off-label use of the treatments. The experts acknowledged that it may be easier to demonstrate a two-step reduction in the SUN score in children with severe uveitis compared with those with mild uveitis. Children entering the study with 1 + cells in the anterior chamber would need to achieve a cell score of 0 to show a meaningful reduction in cellular activity, and this tempered the experts’ expectation of treatment efficacy.

The use of a Bayesian clinical trial design allows the consensus information to be combined with the data of a future clinical trial. To ensure that decisions about the potential benefit of secukinumab as a potential treatment for JIA-associated uveitis would not solely be based on opinion, we now examine the strength of evidence coming from the elicited prior distributions compared to the planned trial size of 20 patients per arm.

One metric that is useful in this context is the effective sample size (ESS). This is the number of observations necessary to obtain the prior distributions displayed in Fig. 2, had one not had any information about the treatment effect beforehand (i.e. starting with a uniform distribution). As both the prior distributions are modelled as beta distributions, one can obtain the ESS analytically as the sum of the hyperparameter of these distributions (Table 1 Parameters of the consensus beta prior distributions in the Appendix).

The ESS for the consensus prior distribution of adalimumab therefore is 15.7, while for secukinumab is 13.1. As expected, the level of information about adalimumab for treatment of JIA-associated uveitis is higher than that available for secukinumab. At the same time, the total amount of information provided by the consensus prior distribution is lower than the planned sample size of 20 patients per treatment arm for the RCT itself. Consequently, the decisions based on the combined evidence would still be driven by the trial data, yet substantial enhancement of the power of the study can be expected by adding information from the equivalent of almost 30 patients.

## Conclusions

The Bayesian analysis adds substantial enhancement of the power of the study and supports a trial of adalimumab and secukinumab for JIA-associated uveitis and chronic anterior uveitis.

## Electronic supplementary material

Below is the link to the electronic supplementary material.

Supplementary Material 1: Structured questionnaire

## Data Availability

The datasets during and/or analysed during the current study available from the corresponding author on reasonable request.

## Appendix

**Table 1:** Parameters of the consensus beta prior distributions

Beta parameter	Original response criterion	Revised response criterion
	**Adalimumab**	
α	10.16	15.99
β	5.512	7.123
	**Secukinumab**	
α	7.13	8.43
β	6.015	5.953

## Abbreviations

ADA: Adalimumab
CNO: Chronic Non–Bacterial Osteomyelitis
CRMO: Chronic Recurrent Multi–Focal Osteomyelitis
EudraCT: European Union Drug Regulating Authorities Clinical Trials Database
ESS: Effective Sample Size
ISRCTN: International Standard Randomised Controlled Trial Number
MTX: Methotrexate
JIA: Juvenile Idiopathic Arthritis
RCT: Randomised Controlled Trial
SEC: Secukinumab
SUN: Standardisation of Uveitis Nomenclature
UK: United Kingdom
