# Supplementary material for: Bayesian trial of adalimumab versus secukinumab for children with juvenile idiopathic arthritis associated uveitis or chronic anterior uveitis
Source: Pediatr Rheumatol Online J. 2025 May 19;23:55. doi: 10.1186/s12969-025-01107-1 (PMC12090588; doi:10.1186/s12969-025-01107-1)
Supplement: Supplementary file 1 — Supplementary Material 1: Structured questionnaire [file 12969_2025_1107_MOESM1_ESM.pdf]

Name of Expert: Name of Statistician:

The following questions are to be answered before meeting with the statistician. Below questions regarding efficacy of the two treatments in question, adalimumab and secukinumab. We remind you not to consult with fellow clinicians regarding answers to these questions. Consultation time will be given to arrive at a conclusion which can be agreed upon.

Treatment regimens:

1) Adalimumab (20mg/0.8ml for patients <30kg or 40mg/0.8ml for patients weighting >30kg, s/c injection every 2 weeks based on body weight)
2) weekly secukinumab injections (75mg if weighing less than 25kg, 150mg if weighing between 25kg and less than or equal to 50kg or 300mg if weighing more than 50kg weekly for the first 4 weeks and then every 4 weeks thereafter).

*All patients will continue with their normal methotrexate (MTX) or mycophenolate (MMF).*

**NOTE:** Please provide answers to the questions overleaf assuming patients are offered these treatment options in the doses stated.

Questions regarding proportions of successful treatment are presented on Page 2. Treatment success for a patient (scoring 1 for the primary endpoint) is defined as the following:

**Success is defined by a patient obtaining a 2 step decrease in the level of inflammation (anterior chamber cells) or decrease to zero between baseline (prior to trial treatment initiation) and after 12 weeks of treatment (as per SUN criteria).**

The 'Final answer' column is to be filled in with the assigned statistician once you are satisfied your answers characterise what your opinion is.

The following are questions regarding efficacy of the treatments.

| | Initial answer | Final answer |
|---|---|---|
| Assuming the patient tolerates ADA, what do you think the 12 week response rate for children with JIA treated with ADA in combination with MTX or MMF is? | | |
| Assuming the patient tolerates ADA, provide a proportion such that you are 75% sure (25th percentile) that the true 12 week response rate on ADA plus MTX or MMF exceeds this value. | | |
| Assuming the patient tolerates the drugs, what is the chance that the 12 week response rate on SEC plus MTX or MMF is higher than that on ADA plus MTX or MMF? | | |
| Assuming the patient tolerates the drugs, what is the chance that the 12 week response rate on ADA plus MTX or MMF exceeds that on SEC plus MTX or MMF by more than 10%? | | |
| Assuming the patient tolerates the drugs, what do you think the 12 week response rate on SEC plus MTX or MMF is? | | |
| Assuming the patient tolerates the SEC, provide a proportion such that you are 75% sure (25th percentile) that the true 12 week response rate on SEC plus MTX or MMF exceeds this value. | | |
